# Central and East European migrant men who have sex with men in London: a comparison of recruitment methods

**DOI:** 10.1186/1471-2288-11-69

**Published:** 2011-05-17

**Authors:** Alison R Evans, Graham J Hart, Richard Mole, Catherine H Mercer, Violetta Parutis, Christopher J Gerry, John Imrie, Fiona M Burns

**Affiliations:** 1 Centre for Sexual Health and HIV Research, Research Department of Infection and Population Health, University College London, London, UK; 2 The School of Slavonic & East European Studies, University College London, London, UK; 3 The Africa Centre for Health & Population Studies, University of Kwazulu-Natal, Mtubatuba, South Africa

## Abstract

**Background:**

Following the expansion of the European Union, there has been a large influx of Central and East European (CEE) migrants to the UK. CEE men who have sex with men (MSM) represent a small minority within this population that are none-the-less important to capture in sexual health research among the CEE migrant community. This paper examines the feasibility of recruiting CEE MSM for a survey of sexual behaviour in London using respondent driven sampling (RDS), via gay websites and in GUM clinics.

**Methods:**

We sought CEE MSM to start RDS chain referral among GUM clinic attendees, our personal contacts and at gay events and venues in central London. We recruited CEE MSM (n = 485) via two popular websites for gay men in Britain (March-May 2009) and at two central London GUM clinics (n = 51) (July 2008-March 2009).

**Results:**

We found seventeen men who knew other CEE MSM in London and agreed to recruit contacts into the study. These men recruited only three men into the study, none of whom recruited any further respondents, and RDS was abandoned after 7 months (July 2008-January 2009). Half of the men that we approached to participate in RDS did not know any other CEE MSM in London. Men who agreed to recruit contacts for RDS were rather more likely to have been in the UK for more than one year (94.1% vs 70.0%, p = 0.052). Men recruited through gay websites and from GUM clinics were similar.

**Conclusions:**

The Internet was the most successful method for collecting data on sexual risk behaviour among CEE MSM in London. CEE MSM in London were not well networked. RDS may also have failed because they did not fully understand the procedure and/or the financial incentive was not sufficient motivation to take part.

## Background

Convenience sampling is a non-probability sampling technique whereby respondents are recruited from a group of individuals that is readily available to the researcher [1]. It is a cost-effective and easy method for recruiting non-representative samples of men who have sex with men (MSM) [2]. Effective convenient sampling techniques among MSM include recruitment from sexual health clinics and gay venues and, more recently, through online surveys which are particularly efficient for reaching large numbers of geographically dispersed MSM [3]. However, convenience sampling is prone to biased estimates because the samples that are recruited are not representative of the population as a whole. Respondent driven sampling (RDS) is an alternative approach that was developed to recruit unbiased samples of hard to reach populations [4,5].

RDS is especially useful for HIV surveillance [6] and has been used successfully to recruit representative samples of MSM throughout the world [7-9]. Members of the target group are selected by the research team and, having completed the survey, they invite up to three peers from their social network who are also members of the target group to participate in the study. These three peers are asked to complete the survey and then recruit three more contacts and so on until the sample size is reached. Bias is minimised through the production of long recruitment chains whereby the sample is no longer dependent on the non-randomly selected initial participants. Respondents are given an incentive both for completing the questionnaire and for each respondent successfully recruited into the study.

Convenience sampling through online surveys is now well established as a means for recruiting MSM, [10-13] but there is little published research comparing Internet sampling among MSM with the use of RDS. In one notable exception, RDS was adopted as a recruitment strategy among MSM in Estonia where it failed to achieve the required sample size and reached a less diverse sample of MSM than the Internet arm of the study [14].

The accession of ten Central and East European (CEE) countries (Bulgaria, Czech Republic, Estonia, Hungary, Latvia, Lithuania, Poland, Romania, Slovakia and Slovenia) to the European Union since May 2004 resulted in a large influx of predominantly young economic migrants to the UK [15]. Homosexuality is no longer a criminal offence in CEE countries but stigmatization and discrimination are widely reported in the region [16,17]. The stigma associated with homosexuality may impact upon the sexual knowledge and behaviours of CEE MSM, as well as their willingness to access sexual health services [18]. We therefore set out to examine the extent to which sexual risk behaviours are practised by CEE migrant MSM in the UK, as part of the SALLEE project (Sexual attitude and lifestyle of London's East Europeans).

We initially selected RDS as a potentially expeditious means for accessing CEE MSM in London because they form a minority, hidden group within the CEE migrant community that is likely to be at risk for acquisition and transmission of sexually transmitted infection but would be difficult to reach by other means. Although the CEE region is made up of heterogeneous populations, CEE migrants in London form networks with people from their own and other CEE countries, [19] which indicates that RDS would be an appropriate sampling strategy. While London also provides many opportunities for MSM to meet one another, we are not aware of any other studies that have used RDS to recruit a sub-population of MSM in this or any other part of the UK.

Our study of sexual behaviour among CEE MSM in London is part of a wider survey of sexual behaviour among migrants in London from the ten CEE countries which have joined the European Union since May 2004. In this paper, we examine the feasibility of strategies for recruiting CEE MSM who represent an important sub-sample within the minority population of CEE migrants in London. We include a GUM clinic sample of CEE migrants attending two GUM services in central London and samples which specifically targeted CEE MSM through RDS and gay websites.

## Methods

### Social mapping

We conducted an extensive social mapping exercise in order to locate venues for recruiting a community sample of CEE migrants in London, as described in detail elsewhere [20]. We identified two London boroughs as suitable areas and recruited men and women from across the CEE region in these boroughs [21]. During this mapping exercise, we did not find any venues where we could specifically target CEE MSM. This suggested that alternative sampling methods were needed for this sub-population.

### GUM clinic sample

Individuals who identified as CEE migrants in two central London GUM clinics were approached and asked to complete a questionnaire on a hand-held computer (July 2008-March 2009) [20]. Recruitment in the clinics was mainly undertaken by two members of the research team, one of whom was a native speaker of Polish and Lithuanian. The days and times of recruitment in the clinics varied over the data collection period. A £5 high street voucher was offered as an incentive.

### Respondent driven sample (RDS)

CEE MSM were sought to act as RDS "seeds" (men who knew other CEE MSM in London to start the chain referral process) among GUM clinic attendees, personal contacts of the research team and at gay events and venues in central London (July 2008-January 2009). Seeds completed the questionnaire on sexual behaviour and were given coupons to invite three other CEE MSM in London to take part. The coupons asked men to telephone the research team to make an appointment with the research team to complete the questionnaire. All men completing the survey as part of the RDS process were offered £10 for completing the questionnaire and a further £5 for each respondent recruited. Each recruit was offered £10 in turn for completing the questionnaire and £5 for each further recruit and so on. To establish the size of respondents' social networks, seeds and recruits were asked the number of CEE MSM in London that they knew: specifically they were asked "*Do you know any other Central or East European gay men in London?*" and if they answered yes, "*How many of these men do you know well enough to talk about this survey with?"*

### Internet sample

Subsequently, we adopted an online strategy for recruiting CEE MSM, as described in detail elsewhere [22]. The questionnaire was posted on the Internet and a link to it was placed on banners which appeared when men were browsing the personal profiles of London men in Gaydar http://www.gaydar.co.uk for 6 weeks (March to April 2009). A link was also placed on the homepage of GayRomeo http://www.gayromeo.com for 4 weeks (April to May 2009). These websites were selected after asking CEE MSM which sites for gay men they used. No incentive was offered for online participation.

### Study instrument

The survey instrument was a self-completed questionnaire that was fielded using hand-held computers for the RDS and GUM clinic samples and a web survey for the Internet sample. It included no information that would allow respondents to be identified. It was available in twelve languages (the ten CEE languages plus English and Russian). The questionnaire concentrated on sexual risk behaviour and, where possible, questions from previously validated questionnaires were used in order to maximize their reliability and validity. It took about ten minutes to complete.

### Statistical analysis

Standard statistical tests including chi-square and Student's t-test were used to examine associations between samples on background characteristics and partner numbers. We compared samples of men who agreed to act as RDS recruiters vs those who did not, who were recruited in a GUM clinic vs on the Internet, and who were recruited on Gaydar vs GayRomeo. Analysis was performed using SPSS12.0 (SPSS Inc, Chicago, Illinois, USA). The analysis includes literate men aged over 17 years who self-identified as CEE migrants, were recruited from venues or lived in London and reported sex with a man in the past five years.

### Ethics

The study was granted approval from the Camden and Islington Community Research Ethics Committee (07/H0722/110).

## Results

A total of 536 CEE MSM in London were recruited (table 1). They came from all ten CEE countries but the largest proportion were from Poland (40.9%). A minority of men spoke more than one CEE language at home when they were growing up (13.5%) and one fifth of men (21.3%) completed the questionnaire in English. The majority of men (90%, 485/536) were recruited via gay websites. The GUM clinic sample included 51 MSM (54% of all CEE men in this sample). We asked 20 MSM in the clinic sample if they would be willing to participate in RDS and 10 agreed (50%). We asked 27 men in the "gay community" (identified via personal contacts or gay events and venues in central London) the same question and 7 agreed (26%). Other attempts to find CEE MSM to act as seeds (through websites for gay men, the gay press, an online group concerned with sexuality in CEE countries) did not result in any responses.

**Table 1:** Place of birth, language spoken at home and language of questionnaire completion among CEE MSM in London

	n = 563	
	***n***	%
**Country of birth**		
Bulgaria	*28*	5.0
Czech Republic	*42*	7.5
Estonia	*16*	2.8
Hungary	*52*	9.2
Latvia	*24*	4.3
Lithuania	*35*	6.2
Poland	*230*	40.9
Romania	*38*	6.7
Slovakia	*55*	9.8
Slovenia	*23*	4.1
More than one CEE language spoken at home ^1^	*76*	13.5
Questionnaire completed in English	*120*	21.3

^1 ^CEE languages (including Russian) reported as spoken at home between ages 10 to 16 years

Among the 78 men recruited in clinics and the gay community, we recorded data from 39 men about whether they knew any other CEE MSM in London and 20 (51%) did. Only 3 of these men reported that they knew other CEE MSM and declined to act as seeds. The 17 men who agreed to recruit other men referred only three men into the study, two of whom declined to recruit any further men and one of whom failed to recruit any further respondents. RDS was therefore abandoned after seven months (July 2008-January 2009).

The 17 recruiters reported networks of CEE MSM ranging from 1 to 26 men, with a mean of 6 and a median of 3 men. Neither of the two men who recruited other men into the study returned to redeem their £5 for each recruit so we were unable to gather any further information about their networks.

We compared those men who agreed and those who refused to act as seeds to recruit CEE MSM in London (table 2). Men who agreed were more likely to have been in the UK for more than one year (94.1% vs 70.0%, p = 0.052). We also compared men recruited using our two other strategies. The only significant difference was that men recruited via gay websites were less likely to belong to a religion than men recruited in GUM clinics (38.5% vs 54.9%, p = 0.023). We compared men recruited via Gaydar to men recruited via GayRomeo. Gaydar men were somewhat older than GayRomeo men (30.1 vs 28.6 years, p = 0.023) and were more likely to have been in the UK for more than one year (89.3% vs 78.9%, p = 0.003). Again, there were no significant differences between the two samples on partner numbers.

**Table 2:** Background characteristics and partner numbers among CEE MSM in London, by sample

	RDS recruiter n = 17		RDS non-recruiter n = 30		p value	GUM clinic n = 51		Gay websites n = 485		p value	Gaydar n = 201		GayRomeo n = 284		p value
	***n***	%	***n***	%		***n***	%	***n***	%						
**Background characteristics**															
Age in years: mean (SD)	27.4 (5.0)		26.0 (4.1)		0.321	27.4 (4.7)		29.2 (7.2)		0.080	30.1 (8.0)		28.6 (6.4)		0.023
Working	*14*	82.4	*25*	83.3	0.932	*44*	86.3	*387*	80.0	0.278	*156*	77.6	*231*	81.6	0.277
Has a degree	*10*	58.8	*16*	53.3	0.716	*34*	66.7	*275*	56.8	0.176	*122*	60.7	*153*	54.1	0.147
Born in A8 country (vs A2 country) ^1^	*16*	94.1	*25*	83.3	0.287	*42*	82.4	*408*	87.7	0.274	*172*	88.7	*236*	87.1	0.610
More than one year in UK	*16*	94.1	*21*	70.0	0.052	*42*	82.4	*397*	83.2	0.874	*176*	89.3	*221*	78.9	0.003
Returned home twice or more (past year)	*8*	47.1	*19*	65.5	0.220	*28*	54.9	*291*	60.2	0.459	*122*	60.7	*169*	59.9	0.865
Belongs to a religion	*9*	52.9	*15*	50.0	0.846	*28*	54.9	*186*	38.5	0.023	*82*	41.0	*104*	36.7	0.344
**Partner numbers**															
One or more new AI partners (past year) ^2^	*14*	87.5	*20*	66.7	0.125	*42*	84.0	*358*	75.1	0.159	*146*	75.3	*212*	74.9	0.932
More than ten male partners in past five years	*11*	64.7	*19*	63.3	0.925	*34*	66.7	*323*	66.6	0.992	*138*	68.7	*185*	65.1	0.419

^1 ^A8 countries joined the European Union in May 2004: Czech Republic, Estonia, Hungary, Latvia, Lithuania, Poland, Slovakia, Slovenia and A2 joined in January 2007: Bulgaria and Romania; ^2 ^AI = anal intercourse

## Discussion

The established networks among CEE migrants in London and the extensive opportunities for networking among gay men in the UK capital suggested that RDS would be an appropriate strategy for recruiting CEE MSM in London. Despite its successful implementation among gay men in other countries, [7-9] however, RDS was unsuccessful among CEE MSM in London. While our findings are limited by small sample sizes and lack of statistical power, they highlight key considerations for the feasibility of RDS among different groups of MSM and particularly among migrant MSM from across the CEE region.

Firstly, we believe the main reason for the failure of RDS in our study was the lack of networking among CEE MSM in London. This also limited the applicability of RDS to sex workers in East Europe [23]. Half the men did not know any other CEE gay men in London. This may be related to homophobia and stigmatisation which are prevalent in CEE countries [16,17] and which may have encouraged their move to the UK [24]. Moving to the UK to avoid homophobia at home was repeatedly cited by CEE MSM in in-depth qualitative interviewees that were also conducted as part of the SALLEE project (R. Mole, manuscript in preparation). Importantly for the success of RDS, such fear of homophobia and stigmatisation may raise concerns among MSM about coming forward and engaging in research on sexual behaviour. Although it is suggested that RDS does not require formative research, [6] our findings support previous research which highlights the importance of exploring social networks and acceptability of RDS among the target group before implementing RDS [25]. Our experience has shown that CEE MSM in London are readily recruited via the Internet and formative research would have been usefully undertaken through a short online survey asking about the number of CEE MSM in London known to respondents, and the willingness of respondents and their peers to take part in face-to-face research of this nature.

Secondly, the majority of CEE MSM were working and may not have been motivated by £5 for each recruit. While the study site was in a convenient central London location, their contacts may not have considered £10 as adequate compensation for the time and effort involved in travelling to the site to complete the questionnaire. The importance of an adequate incentive has been highlighted elsewhere [14,23]. Unfortunately, however, the anonymous nature of RDS means that contact details were not collected and our study is limited by the lack of follow up to establish why men failed to recruit their peers.

Thirdly, the failure of RDS in this study of CEE MSM in London may be related to the language problems associated with a population originating in ten different countries. It was often not possible to language-match fieldworkers with CEE men, who may not therefore have fully understood the procedures for recruiting their peers into the study. Our respondents came from all ten CEE countries and only one fifth chose to complete the questionnaire in English. The instructions on the RDS coupons were in English, however, as it was impractical to issue coupons in all eleven CEE languages and English was likely to be the common language between CEE MSM in London. Although the vast majority of men had been in the UK for more than one year, it is possible that respondents' English language abilities may have had an adverse effect on the implementation of RDS in this study.

By contrast, the Internet was particularly effective for reaching CEE MSM in London. The same was also found to be the case for recruiting MSM in Estonia [14]. Complete anonymity and ease of access to the questionnaire are likely to have facilitated this. In addition, men who responded to the Internet survey could click on a link taking them directly to versions of the questionnaire in eleven CEE languages and English. Although we were unable to verify whether responses were from eligible CEE MSM in London, it is unlikely that respondents lied in order to participate, as there was no monetary incentive to do so. While MSM recruited from gay dating websites are likely to report higher levels of sexual risk behaviour than the general population of MSM, [26] we found that men recruited through these websites were similar in background characteristics and partner numbers to men recruited from GUM clinics. In addition there was little difference between men recruited from the two websites. Although the Gaydar recruits were somewhat older and had been in the UK for a little longer than men from GayRomeo, they reported similar numbers of partners. This provides some confidence in these methods for recruiting CEE MSM in London for surveys of sexual behaviour.

## Conclusions

The Internet succeeded over RDS for collecting data on sexual risk among CEE MSM in London. The Internet sample was similar to the sample of men recruited in GUM clinics, although it is unlikely to be representative of all CEE MSM in London. RDS is only successful to the extent that potential respondents are networked which requires thorough testing before fieldwork. The procedure for recruiting their peers must be clearly explained in language that all respondents can understand. RDS puts the onus on respondents to take a proactive role in participation and in the recruitment of their peers. In this study, incentives for participation and recruitment, such as the financial reward, may have been insufficient to outweigh this burden.

## Competing interests

The authors declare that they have no competing interests.

## Authors' contributions

FB, CM, CG, RM, JI, GH participated in the design of this study. AE, VP and FB were responsible for study coordination and data collection. AE conducted the statistical analysis and was the lead writer of this paper. All authors have contributed to the drafting of the manuscript and have seen and approved the final version.

## Pre-publication history

The pre-publication history for this paper can be accessed here:

http://www.biomedcentral.com/1471-2288/11/69/prepub

## References

[B1] Bryman A Social Research Methods 2004 2 Oxford: Oxford University Press

[B2] McGarrigle CA Fenton KA Gill ON Hughes G Morgan D Evans B Behavioural surveillance: the value of national coordination Sex Transm Infect 2002 11 398 405 10.1136/sti.78.6.398 12473798 PMC1758341

[B3] Hickson F Weatherburn P Reid D Jessup K Hammond G Testing Targets: Findings from the United Kingdom Gay Men's Sex Survey 2007 Sigma Research

[B4] Heckathorn DD Respondent-driven sampling: a new approach to the study of hidden populations Soc Probl 1997 11 174 199 10.1525/sp.1997.44.2.03x0221m

[B5] Salganik MJ Heckathorn DD Sampling and estimation in hidden populations using respondent-drive sampling Sociol Methodol 2004 11 193 239 10.1111/j.0081-1750.2004.00152.x

[B6] Magnani R Sabin K Saidel T Heckathorn D Review of sampling hard-to-reach and hidden populations for HIV surveillance AIDS 2005 11 Suppl 2 S67 S72 15930843 10.1097/01.aids.0000172879.20628.e1

[B7] Malekinejad M Johnston LG Kendall C Kerr LRFS Rifkin MR Rutherford GW Using respondent-driven sampling methodology for HIV biological and behavioural surveillance in international settings: a systematic review AIDS Behav 2008 11 S105 S130 10.1007/s10461-008-9421-1 18561018

[B8] Ramirez-Valles J Heckathorn DD Vázquez R Diaz RM Campbell RT From networks to populations: the development and application of respondent-driven sampling among IDUs and Latino gay men AIDS Behav 2005 11 387 402 10.1007/s10461-005-9012-3 16235135

[B9] Johnston LG Khanam R Reza M Khan SI Banu S Alam MS Rahman M Azim T The effectiveness of respondent driven sampling for recruiting males who have sex with males in Dhaka, Bangladesh AIDS Behav 2008 11 294 304 10.1007/s10461-007-9300-1 17712620

[B10] Hospers HJ Kok G Harterink P De Zwart O A new meeting place: chatting on the internet, e-dating and sexual risk behaviour among Dutch men who have sex with men AIDS 2005 11 1097 101 10.1097/01.aids.0000174457.08992.62 15958842

[B11] Tikkanen R Ross MW Looking for sexual compatibility: experiences among Swedish men in visiting internet gay chat rooms Cyberpsychol Behav 2000 11 605 16 10.1089/109493100420205

[B12] Elford J Bolding G Davis M Sherr L Hart G Web-based behavioral surveillance among men who have sex with men: a comparison of online and offline samples in London, UK J Acquir Immune Defic Syndr 2004 11 421 6 10.1097/00126334-200404010-00012 15097159

[B13] Bull SS McFarlane M Rietmeijer CA HIV and sexually transmitted infection risk behaviors among men seeking sex with other men on-line Am J Public Health 2001 11 988 9 10.2105/AJPH.91.6.988 11392947 PMC1446481

[B14] Johnston LG Trummal A Lõhmus L Ravalepik A Efficacy of convenience sampling through the internet versus respondent driven sampling among males who have sex with males in Tallinn and Harju County, Estonia: challenges reaching a hidden population AIDS Care 2009 11 1195 1202 10.1080/09540120902729973 20024780

[B15] Home Office UK Border Agency: Accession Monitoring Report 2009 Crown copyright http://www.ukba.homeoffice.gov.uk accessed October 2009

[B16] World Health Organisation HIV and other STIs among MSM in the European Region 2008 World Health Organisation http://www.euro.who.int accessed 29 June 2010

[B17] European Commission Eurobarometer 66: Public Opinion in the European Union 2007 European Commission http://ec.europa.eu/public_opinion accessed 29 June 2010

[B18] Quinn S Accessing health: the context and the challenges for LGBT people in Central and Eastern Europe 2006 ILGA Europe http://www.ilga-europe.org accessed 25 February 2011

[B19] Parutis V White, European and Hardworking: East European Migrants' Relationships with Other Communities in London J Baltic Stud 2011 11 2

[B20] Evans AR Parutis V Hart G Mercer CH Gerry CJ Mole R French RS Imrie J Burns FM The sexual attitudes and lifestyles of London's Eastern Europeans (SALLEE Project): design and methods BMC Public Health 2009 11 399 published Online First: 30 October 2009 10.1186/1471-2458-9-399 19878564 PMC2777871

[B21] Burns FM Evans AR Mercer CH Parutis V Gerry CJ Mole RCM French RS Imrie J Hart GJ Sexual and HIV risk behaviour in Central and Eastern European migrants in London Sex Transm Infect 2011 11 318 324 10.1136/sti.2010.047209 21593470

[B22] Evans AR Hart G Mole R Mercer CH Parutis V Gerry CJ Imrie J Burns FM Central and East European migrant men who have sex with men: an exploration of sexual risk in the UK Sex Transm Infect 2011 11 325 30 published Online First: 8 December 2010 10.1136/sti.2010.046409 21147893

[B23] Simic M Johnston LG Platt L Baros S Andjelkovic V Novotny T Rhodes T Exploring barriers to 'respondent driven sampling' in sex worker and drug-injecting sex worker populations in Eastern Europe J Urban Health 2006 11 Suppl 1 6 15 10.1007/s11524-006-9098-6 PMC1705510 17109206

[B24] Delpech VC Yin Z Abernethy J Hill C Logan L Chadborn TR Rice BD The impact in the UK of the Central and Eastern European HIV epidemics Epidemiol Infect 2009 11 1266 e71 10.1017/S0950268809002155 19224655

[B25] Johnston LG Whitehead S Simic-Lawson Kendall C Formative research to optimize respondent-driven sampling surveys among hard-to-reach populations in HIV behavioural and biological surveillance: lessons leaned from four case studies AIDS Care 2010 11 784 792 10.1080/09540120903373557 20467937

[B26] Evans AR Wiggins RD Mercer CM Bolding GJ Elford J Men who have sex with men in Great Britain: comparison of a self-selected internet sample with a national probability sample Sex Transm Infect 2007 11 200 205 published Online First: 29 November 2006 17135330 10.1136/sti.2006.023283 PMC2659092

